# Pulmonary Metastasectomy in Pediatric Solid Tumors

**DOI:** 10.3390/children6010006

**Published:** 2019-01-08

**Authors:** Nicole J. Croteau, Todd E. Heaton

**Affiliations:** Pediatric Surgical Service, Department of Surgery, Memorial Sloan Kettering Cancer Center, New York, NY 10065, USA; croteaun@mskcc.org

**Keywords:** pediatric lung metastases, pediatric solid tumors, pulmonary metastasectomy, management of lung metastases

## Abstract

Metastatic disease and the complications of treating metastatic disease are the primary causes of mortality in children with solid malignancies. Nearly 25% of children with solid tumors have metastatic disease at initial diagnosis and another 20% develop metastases during or after treatment. The most common location of these metastases is the lung. The role of surgery in metastatic disease depends greatly on the histology of the primary. In general, tumors that are refractory to adjuvant therapies are most appropriate for pulmonary metastasectomy. This article will summarize the indications for metastasectomy in pediatric solid tumors and discuss the ongoing debate over the technique of metastasectomy in osteosarcoma.

## 1. Introduction

Treatment of pediatric solid tumors has significantly improved in the last several decades, with overall survival rates of 75–90% in non-metastatic tumors. However, there still exists a large percentage of children who present with metastatic solid tumors (10–30%) and another 15–20% who have distant site relapses [1]. While there have been significant advances in treatment of non-metastatic disease, children with metastatic tumors still have poor prognoses, with overall survival ranging widely from 20% to 70% depending on histology [1]. Systemic therapy is the mainstay of treatment in patients with metastases as this is a disseminated process. However, surgical management can be therapeutic. Children with malignancies that respond poorly to systemic therapy are more likely to have a beneficial response to surgical resection than those patients who have tumors that respond well to adjuvant therapy. While metastases can occur at a number of sites, such as lymph nodes, bone, liver, and brain, the majority of metastases in pediatric solid tumors occur in the lungs. In this review, we will discuss the surgical treatment of pulmonary metastases, current diagnosis and localization techniques, survival in a variety of solid tumors, and the special case of metastatic osteosarcoma.

## 2. Historical Perspective

In 1961, Richardson found a 5-year survival rate of 23% in 35 patients who underwent pulmonary metastasectomy [2]. Since then, many publications have addressed management principles for pulmonary metastasectomy, finding that: (1) staged bilateral resections are well-tolerated, (2) accurate diagnosis can help avoid unnecessary toxic therapy, (3) number of metastases and disease-free interval are not contraindications to metastasectomy, and (4) histology is very important [3,4]. In 1969, Kilman et al. popularized the wedge resection, which allowed multiple bilateral lung lesions to be resected while preserving maximal lung function [5]. Because most published series from 1960 to 2000 grouped multiple histologies together, analysis of outcome after pulmonary metastasectomy was impossible [2,3,4,5,6]. Some studies avoided this problem by focusing only on one histology [7], and some shed light on this problem by showing the difference in outcomes between histologies [8]. In recent decades, additional literature has brought new insights to metastasectomy in pediatric solid malignancies.

## 3. Diagnosis and Localization

A prospective study by Chang et al. in 1979 demonstrated higher sensitivity of computed tomography (CT) scan over plain radiographs for identifying pulmonary nodules, which made CT scanning the new gold standard [9]. As technology has continued to improve over time, CT has become even more sensitive and is still the favored method of identification and surveillance of pulmonary nodules in children. There are, however, limitations to CT in pediatric solid tumors, including the fact that, while highly sensitive, CT lacks specificity and cannot differentiate benign from malignant nodules, leading to false-positive interpretations. This can ultimately lead to increased anxiety, unnecessary surgery, and the possibility of over-treatment if a biopsy is not performed.

In 1992, Rosenfield et al. reported that out of 13 CT-identified lung nodules in pediatric patients, less than half were malignant [10]. Similarly, in 2006, after years of advancing technology, McCarville et al. found that 42% of children with CT-identified lung nodules had benign lesions at biopsy. Interestingly, when CT scans were independently reviewed, malignant lesions were correctly identified only 57–67% of the time, with poor agreement between reviewers [11]. While this is true of most pediatric malignancies, the sensitivity of pre-operative CT has been questioned the most in osteosarcoma where CT has been shown to underestimate the total number of metastases in up to 35% of cases when compared to the number of lesions found by palpation [11,12,13,14]. Despite the deficiencies of CT, it is the gold standard for identifying pulmonary metastases in pediatric solid tumors. The calcified metastases in osteosarcoma allow for identification by palpation not possible in non-calcified histologies, leading to specific issues discussed later in the chapter.

After CT identification of a pulmonary nodule, further difficulties may arise in localizing the nodule for diagnostic or therapeutic resection. Superficial lesions can be seen on visual inspection intraoperatively and larger, firmer lesions can be palpated, but smaller, softer, and/or deeper lesions can easily be missed. Because the goal of metastasectomy is the total resection of metastases while maintaining maximal normal lung tissue, resorting to lobectomies or segmentectomies is counterproductive. Many techniques, including pre-operative marking with wires, coils, and dyes, and localization with intraoperative ultrasound, have been used in an attempt to solve this problem [15,16,17,18]. While all of these techniques can be useful, they all have drawbacks; dyes can spread along the pleura, while coils and wires can be inaccurately placed or dislodged, and intraoperative ultrasound can be limited by the depth of the lesion and the amount of air in the lung, allowing a success rate of only 80–90% for all techniques. One recent study using technetium-99 macro-aggregated albumin localization attempted localization of 24 nodules in 11 patients over a 2-year period and was successful in 23 [19]. Hopefully, this is the harbinger that new, more successful techniques can be developed, but current techniques are imperfect.

The management of pulmonary metastases is highly dependent on the histology of the primary tumor, with two general groups emerging: (1) tumors that respond well to systemic adjuvant therapy and (2) tumors that respond poorly to systemic adjuvant therapy. We will address each of these two groups and then discuss the special case of osteosarcoma.

## 4. Tumors Responsive to Adjuvant Therapy

### 4.1. Wilms Tumor

While the overall survival rates in patients with Wilms tumor are high, the 10% of patients who present with pulmonary metastases have a significantly worse prognosis [3,8,20]. These nodules are generally treated with whole-lung radiation with excellent outcomes, but this has been associated with interstitial lung disease (5–12% incidence within 15 years) and breast cancer (15% risk by age 40 in female survivors) [21,22,23,24]. In the past, pulmonary lesions in Wilms tumor patients were only treated if they could be seen on plain film. However, as CT scans emerged as more sensitive (though not specific), the question of treating patients with pulmonary nodules only seen on CT arose. In studies between the late 1980s and early 2000s, no significant overall survival difference was found between patients with CT-only pulmonary nodules and those without lung metastases [25,26,27], which was attributed to the increased diagnosis of benign lung disease. In 2012, however, the National Wilms Tumor Study (NWTS)-4 and -5 found an improved 5-year event-free survival (80% vs. 56%, *p* = 0.004) in patients with CT-only nodules treated with three-drug vs. two-drug chemotherapy, but no significant difference in overall survival [28]. A separate analysis of NWTS-5 reported that 26% of patients with biopsied CT-only pulmonary lesions had benign nodules and required no additional treatment [29], emphasizing the importance of differentiating between metastases and benign nodules.

The most recent Children’s Oncology Group (COG) renal tumors trial, AREN0533, eliminated lung radiation for patients who achieved complete lung nodule response following initial chemotherapy. After six weeks of therapy, patients were divided into rapid complete responders (RCRs), who had achieved complete pulmonary remission after 6 weeks of three-drug treatment, and slow incomplete responders (SIRs) if they had not. RCRs then continued receiving chemotherapy and avoided lung radiation, but SIRs received lung radiation and more intensive chemotherapy. The 4-year event-free survival (EFS) and overall survival (OS), of 79.5% and 96.1% for RCRs and 88.5% and 95.4% for SIRs, respectively, were not significantly different, showing that more intensive treatment can be avoided in RCRs [30].

The use of CT scans has clearly increased diagnostic sensitivity but not improved specificity. With the increasing potential for patients to avoid more toxic therapy if their lung nodules are proven benign, the diagnostic value of metastasectomy in Wilms has increased greatly. This has pushed the COG and recent studies to encourage diagnostic biopsy of lung nodules in patients with Wilms tumor and pulmonary lesions that do not respond to initial therapy.

### 4.2. Hepatoblastoma

The survival rate for the 20% of hepatoblastoma patients who present with pulmonary metastases is only 25–50%, which is much lower than patients without metastases [31,32]. While early case reports of metastasectomy for cure and initial chemotherapy trials with chemotherapy alone both showed promise, two larger Japanese trials showed the benefit of a combined approach and emphasized metastasectomy following chemotherapy for residual lung disease [33,34]. This combination of chemotherapy with metastesectomy for residual disease is still used in hepatoblastoma cooperative trials.

In a COG study of 38 patients with hepatoblastoma lung metastases at diagnosis, 9 patients underwent metastasectomy; 8 patients survived and 3 of these developed pulmonary recurrences [32]. The International Society of Pediatric Oncology Liver Tumor Study Group (SIOPEL)-1 study included 22 patients who had lung metastases at diagnosis, seven of whom underwent metastasectomy resulting in four long-term survivors [31]. In another SIOPEL study of 59 patients with relapsed hepatoblastoma, 27 patients (46%) had pulmonary progression of disease and 31 patients (52%) achieved a second remission, with 3-year EFS and OS of 34% and 43%, respectively [35]. A recent multi-institutional review identified 10 pediatric patients with lung-only hepatoblastoma recurrence who underwent metastasectomy, with eight survivors at last follow-up [36].

Complete surgical resection of primary hepatoblastoma and residual metastatic disease is essential for long-term survival. Additionally, PRETEXT III and IV patients who require liver transplantation must have resection of all residual lung disease prior to local control due to the need for post-transplant immunosuppression [4]. The presence of uncontrolled disease at the primary site and the inability to achieve a complete resection while maintaining adequate lung function are absolute contraindications to metastasectomy in hepatoblastoma.

### 4.3. Ewing Sarcoma

The utility of surgery in Ewing sarcoma is difficult to assess because this tumor is very chemo- and radio-sensitive. The conflicting results of early case series made the benefit of metastasectomy unclear [8,37,38,39]. In a 2011 series of 31 patients with Ewing sarcoma with lung metastases, the eight patients who underwent pulmonary metastasectomy had a 5-year survival of 80%, while those who received radiation and/or chemotherapy without surgery had a 0% survival [40]. As with earlier studies, selection bias may have played a role given that the authors did not report or control for disease burden and did not explain their decision regarding choice of therapy.

A more recent study examined 38 Ewing sarcoma patients with isolated pulmonary metastases treated with modern multi-modal therapy between 2000 and 2014. Twenty patients underwent metastasectomy and six received pulmonary radiation postoperatively. Patients were divided into groups by disease burden: Group 1 = solitary nodule <0.5 cm or multiple <0.3 cm, Group 2 = solitary nodule 0.5–1 cm or multiple 0.3–0.5 cm, and Group 3 = solitary nodule >1cm or multiple >0.5 cm; patients were reclassified after six cycles of chemotherapy. Improvements in pulmonary disease burden were seen in 15 patients, with 11 having complete resolution of lung nodules. Metastasectomy was performed following chemotherapy. Viable disease was found in all of Group 3 patients and 63% of Group 2 patients, while Group 1 patients had no viable disease. An improved EFS was seen in patients who had a radiographic response to chemotherapy, but metastasectomy had no effect on survival [41].

The conflicting reports regarding pulmonary metastasectomy in Ewing sarcoma shed little light on its therapeutic benefit. It can still play an important diagnostic role, however, given that the rate of negative biopsy in patients with small to moderate lung nodules is 47%, potentially avoiding additional therapy or radiation in many patients.

### 4.4. Neuroblastoma

In patients with neuroblastoma, pulmonary metastases are rare, with an incidence of 3.6% most recently reported by the International Neuroblastoma Risk Group Study [42]. Prior studies had reported the incidence as between 0.4% and 3.2% [43,44]. However, dedicated lung imaging is generally not obtained in the majority of newly diagnosed neuroblastoma patients. Therefore, all of these may underestimate the actual incidence of pulmonary metastases in patients with neuroblastoma. Patients older than 1 year at diagnosis and those with *MYCN* amplification (high-risk group) have a higher likelihood of metastases in general and lung metastases specifically [42], and patients with lung metastases are more likely to have metastases to other sites [45]. In patients with metastatic neuroblastoma, surgery should be reserved for diagnosis, regardless of the burden or location of metastases. Biopsy of the most easily accessible site is recommended for initial diagnosis or recurrence.

### 4.5. Rhabdomyosarcoma

Patients with rhabdomyosarcoma (RMS) lung metastases are 35 times more likely to have a pulmonary relapse than patients with other types of sarcoma lung metastases [37]. This grim outcome has been confirmed by other reports [46]. In 2004, a European study evaluated 174 RMS patients with metastases, in which 55% had metastases to multiple organ systems. Independent, unfavorable risk factors included age <1 or >10 years, bone or bone marrow involvement, and unfavorable primary site. OS in patients with 0 or 1 of these factors was 47%, while patients with two or more of these factors had an OS of only 9% [47]. A COG study in 2005 found that of patients with metastatic RMS, about 16% have lung only metastases and these patients are more likely to have favorable histology, negative nodes, and parameningeal primaries. Patients with metastases at two or more sites had significantly worse outcomes. Interestingly, few patients with lung only metastases underwent biopsy and 35% did not receive lung radiation even though it is associated with lower rates of lung recurrence [48]. In 2016, a COG report found a 3-year EFS of 69% for patients with 0 or 1 risk factor and a 3-year event-free survival of only 20% for patients with two or more risk factors [49].

Metastasectomy for RMS should be reserved for diagnosis because of the poor outcome and generally good response to chemotherapy and radiation.

## 5. Tumors Less Responsive to Adjuvant Therapy

### 5.1. Non-Rhabdomyosarcoma Soft Tissue Sarcoma (NRSTS)

Included in this group of sarcomas are alveolar soft part sarcoma, synovial sarcoma, chondrosarcoma, and malignant fibrous histiocytoma. These tumors are generally resistant to chemotherapy and radiation and tend to metastasize to the lungs. Because they are so rare, they are difficult to study. Given that this group of tumors is generally resistant to other treatments, metastasectomy is recommended if complete resection is possible [50,51]. As with pulmonary metastases in other histologies, CT scan is the standard for diagnosis and localization techniques are advisable for deeper lesions given that these tumors are soft and difficult to palpate.

In 2006, Kayton et al. found that of 20 patients with alveolar soft part sarcoma, seven patients had lung metastases at diagnosis and 14 (70%) had lung metastases at some point in their course. The 5-year overall survival, however, was 83%, showing the slow rate of progression even with the high rate of pulmonary spread. Because of this, the authors recommended liberal use of metastasectomy [52]. Similarly, a study in Beijing of 64 patients found that 56 of them (88%) developed lung metastases at some point; overall survival for patients with lung metastases was 64%, while those without lung metastases had an overall survival of 95% [53].

Compared to other sarcomas in this group, synovial sarcoma has an unusually good response to chemotherapy, but complete resection is still required for cure. Metastases are present at diagnosis or develop later in 40% of patients, and 80% of these metastases are pulmonary [54,55]. A study in England of 150 patients found an overall survival of 6% 5 years after diagnosis of metastasis, but this increased to 23% in patients who underwent metastasectomy [55]. In 2013, Stanelle et al. found that of 41 patients with metastatic synovial sarcoma, no patients survived 2 years without metastasectomy. With metastasectomy, the 5-year overall survival was 24% [56]. As in other sarcomas in the NRSTS group, patients with metastatic synovial sarcoma should undergo metastasectomy if all metastases can be completely resected.

### 5.2. Adrenocortical Carcinoma

Adrenocortical carcinoma is a rare tumor and is resistant to chemotherapy and radiation. In 2016, in one of the largest cohorts to date, 111 pediatric cases from a national database over 13 years were analyzed. Long-term survival was found to be significantly associated with age, tumor size, extension of tumor, metastatic disease, and margin status [57]. While there are no case series examining the effect of pulmonary metastasectomy in the pediatric population, the adult literature reports its benefit for long-term survival [58,59]. Similarly, case reports in the pediatric population show that metastasectomy can produce long-term survival [60,61]. While the data is limited, adrenocortical carcinoma is a resistant tumor and therefore, pulmonary metastasectomy should be performed whenever complete resection is possible. Additionally, the adult literature shows that these tumors are at high risk of rupture during excision, leading to spillage and ultimately to implants and carcinomatosis. Because of this, minimally invasive techniques, while not contraindicated, should be employed cautiously.

### 5.3. Osteosarcoma

There are 400 new cases of osteosarcoma per year in the United States, 20% of which present with metastasis at diagnosis, and another 22% of which develop metastasis at some point, with 85% of these metastases found in the lung. Overall survival for metastatic osteosarcoma remains poor (17–34%) despite significant advances in the treatment of localized osteosarcoma [62,63,64]. Because of the relatively high incidence of osteosarcoma with lung metastasis and its unresponsiveness to chemotherapy and radiation, there is copious evidence that complete resection of all primary and metastatic disease is essential for long term survival [62,63,64,65,66,67,68,69,70,71,72,73,74,75,76,77]. Improved survival in metastatic osteosarcoma has been associated with lung-only metastasis, fewer metastatic lesions, longer disease-free interval between treatment and metastatic relapse, and better histologic response to chemotherapy [62,64,68,70,72,73,74,77,78,79]. Multiple repeat thoracotomies for subsequent lung-only relapses have been shown to afford some chance of cure, and survivors of multiple metastasectomies generally experience only mild long-term decrease in pulmonary function [37,73,78,80].

Metastasectomy in osteosarcoma, therefore, should be attempted whenever complete surgical resection of the primary and metastatic sites is possible. Miliary disease and/or hilar node or chest wall involvement are relative contraindications, depending on whether all lesions can be resected while maintaining adequate pulmonary function. Extrapleural resection or pneumonectomy may be necessary to clear all disease, but careful pre-operative planning is essential. Patients with metastatic disease and synchronous local recurrence or extra-pulmonary metastasis should only undergo surgery if complete resection of all known disease is possible.

Clearly any lesion visible on CT should be resected when possible; however, the ongoing controversy in the surgical treatment of osteosarcoma lung metastasis is predicated on the lesions that cannot be seen on CT. Despite the sensitivity of CT, multiple studies have shown that pre-operative CT underestimates the total number of metastases found on open exploration in up to 35% of patients [4,11,12,13,14]. In fact, there is evidence that modern pre-operative CTs still miss viable osteosarcoma metastases found by open palpation in a quarter of patients, and up to 60% of patients with unilateral CT lesions have palpable contralateral metastases at exploration [12,81,82]. Presumptively, the ability to find metastases down to 1 mm or less in size is predicated on the production of osteoid by the lesions, forming a grain of sand able to be palpated between the surgeon’s fingers at open exploration. It is this characteristic palpability of lesions down to <1mm that separates osteosarcoma from the histologies previously described and sparks the debate over minimally invasive versus open metastasectomy. Minimally invasive metastasectomy is widely accepted for the histologies previously discussed, because its only limitation is the ability to localize the lesions seen on CT. However, evidence that additional lesions can be found by open palpation in osteosarcoma leads to debate over the effect these small lesions have on outcome and the possibility that they may not be found and removed using minimally invasive techniques. While the need for resection of all visible disease is not in question in osteosarcoma, it has never been proven that resecting these “invisible” lesions increases survival. However, resecting these lesions may have both an unsubstantiated benefit and a potential impact of quality of life and length of stay. However, there is insufficient data to recommend one technique over another, or understand the impact of exploratory contralateral thoracotomy.

Several studies have attempted to address the need for contralateral thoracotomy in patients who present with unilateral metastases on CT. Karplus et al. analyzed 81 patients with unilateral early relapse on CT (<2 years off treatment) who underwent unilateral thoracotomy and found no significant difference between ipsilateral relapse (16%) and contralateral relapse (23%, *p* = 0.18) after a mean follow-up of 2 years [83]. In contrast, Su et al. found that six of eight (75%) unilateral, early relapse patients had contralateral disease on staged contralateral exploration [82]. Fernandez-Pineda et al. reported 16 early and late relapse patients presenting with a single pulmonary nodule who had unilateral thoracotomy, and there was no significant difference in ipsilateral second relapse (3 of 11) and contralateral (7 of 11). However, all 10 early relapse patients had a second relapse, 7 contralaterally, and 2 recurred contralaterally within 2 months. Only one of six late relapse patients had a second relapse [84]. The low likelihood of second relapse in late relapse patients and patients with solitary nodules has been confirmed by other larger studies [85], so the risk of forgoing the contralateral exploration would be less. However, further prospective studies are needed to truly settle this controversy.

The larger unanswered question is the application of minimally invasive surgery to osteosarcoma metastasectomy. Thoracoscopy has made lung surgery much more tolerable for the patient, but it does not allow direct palpation of the lung, and is, therefore, more dependent on the imperfect pre-operative imaging and localization discussed previously. In the study by Fernandez-Pineda et al. of 16 patients with a single pulmonary nodule at relapse no additional nodules were found during ipsilateral thoracotomy [84]. However, less than 10% of osteosarcoma relapses present with a single nodule, and, as noted above, up to a quarter of patients have viable metastatic lesions found at open exploration that are not seen on CT [12,81,82]. The survival implications of these “invisible” lesions remain unclear, and other subtle differences between the open and thoracoscopic approach also merit consideration including, the relative ease of re-exploration after thoracoscopy, and the ability to resect much less normal lung with the open approach. Larger retrospective, and even prospective randomized trials may be necessary to settle this controversy.

## 6. Conclusions

Survival rates for children with metastatic solid tumors have not improved to the same extent as those of patients with localized disease. Although management of metastatic disease relies heavily on systemic therapies, surgery plays an important role. In some cases, the surgery is therapeutic, and in some it plays a diagnostic role and guides further systemic treatment. In general, the more resistant a particular histology is to systemic treatment the more central a role metastasectomy plays in cure. Hopefully, further studies can improve the outcomes of patients with these difficult problems and settle some of the remaining surgical debates surrounding metastasectomy.

## References

[1] Fuchs J., Seitz G., Handgretinger R., Schäfer J., Warmann S.W. (2012). Surgical treatment of lung metastases in patients with embryonal pediatric solid tumors: An update. Semin. Pediatr. Surg..

[2] Richardson W.R. (1961). Progress in pediatric cancer surgery. Recent advances in the surgical management of neoplasms in infants and children. Arch. Surg..

[3] Cliffton E.E., Pool J.L. (1967). Treatment of lung metastases in children with combined therapy. Surgery and/or irradiation and chemotherapy. J. Thorac. Cardiovasc. Surg..

[4] Heaton T.E., Davidoff A.M. (2016). Surgical treatment of pulmonary metastases in pediatric solid tumors. Semin. Pediatr. Surg..

[5] Kilman J.W., Kronenberg M.W., O’Neill J.A., Klassen K.P. (1969). Surgical resection for pulmonary metastases in children. Arch. Surg..

[6] Torre W., Rodriguez-Spiteri N., Sierrasesumaga L. (2004). Current role for resection of thoracic metastases in children and young adults—Do we need different strategies for this population?. Thorac. Cardiovasc. Surg..

[7] Martini N., Huvos A.G., Miké V., Marcove R.C., Beattie E.J. (1971). Multiple pulmonary resections in the treatment of osteogenic sarcoma. Ann. Thorac. Surg..

[8] Heij H.A., Vos A., de Kraker J., Voûte P.A. (1994). Prognostic factors in surgery for pulmonary metastases in children. Surgery.

[9] Chang A.E., Schaner E.G., Conkle D.M., Flye M.W., Doppman J.L., Rosenberg S.A. (1979). Evaluation of computed tomography in the detection of pulmonary metastases: A prospective study. Cancer.

[10] Rosenfield N.S., Keller M.S., Markowitz R.I., Touloukian R., Seashore J. (1992). CT differentiation of benign and malignant lung nodules in children. J. Pediatr. Surg..

[11] McCarville M.B., Lederman H.M., Santana V.M., Daw N.C., Shochat S.J., Li C.S., Kaufman R.A. (2006). Distinguishing benign from malignant pulmonary nodules with helical chest CT in children with malignant solid tumors. Radiology.

[12] Kayton M.L., Huvos A.G., Casher J., Abramson S.J., Rosen N.S., Wexler L.H., Meyers P., LaQuaglia M.P. (2006). Computed tomographic scan of the chest underestimates the number of metastatic lesions in osteosarcoma. J. Pediatr. Surg..

[13] Parsons A.M., Detterbeck F.C., Parker L.A. (2004). Accuracy of helical CT in the detection of pulmonary metastases: Is intraoperative palpation still necessary?. Ann. Thorac. Surg..

[14] Fuchs J., Seitz G., Ellerkamp V., Dietz K., Bosk A., Müller I., Warmann S.W., Schäfer J.F. (2008). Analysis of sternotomy as treatment option for the resection of bilateral pulmonary metastases in pediatric solid tumors. Surg. Oncol..

[15] Waldhausen J.H., Shaw D.W., Hall D.G., Sawin R.S. (1997). Needle localization for thoracoscopic resection of small pulmonary nodules in children. J. Pediatr. Surg..

[16] Parida L., Fernandez-Pineda I., Uffman J., Davidoff A.M., Gold R., Rao B.N. (2013). Thoracoscopic resection of computed tomography-localized lung nodules in children. J. Pediatr. Surg..

[17] Partrick D.A., Bensard D.D., Teitelbaum D.H., Geiger J.D., Strouse P., Harned R.K. (2002). Successful thoracoscopic lung biopsy in children utilizing preoperative CT-guided localization. J. Pediatr. Surg..

[18] Gow K.W., Saad D.F., Koontz C., Wulkan M.L. (2008). Minimally invasive thoracoscopic ultrasound for localization of pulmonary nodules in children. J. Pediatr. Surg..

[19] Polites S.F., Fahy A.S., Sunnock W.A., Potter D.D., Klinkner D.B., Moir C.R., Shen K.R., Ishitani M.B. (2018). Use of radiotracer labeling of pulmonary nodules to facilitate excisional biopsy and metastasectomy in children with solid tumors. J. Pediatr. Surg..

[20] Green D.M., Breslow N.E., Ii Y., Grundy P.E., Shochat S.J., Takashima J., D’Angio G.J. (1991). The role of surgical excision in the management of relapsed Wilms’ tumor patients with pulmonary metastases: A report from the National Wilms’ Tumor Study. J. Pediatr. Surg..

[21] Bond J.V., Martin E.C. (1976). Pulmonary metastases in Wilms’ tumour. Clin. Radiol..

[22] Green D.M., Lange J.M., Qu A., Peterson S.M., Kalapurakal J.A., Stokes D.C., Grigoriev Y.A., Takashima J.R., Norkool P., Friedman D.L. (2013). Pulmonary disease after treatment for Wilms tumor: A report from the national wilms tumor long-term follow-up study. Pediatr. Blood Cancer.

[23] Green D.M., Finklestein J.Z., Tefft M.E., Norkool P. (1989). Diffuse interstitial pneumonitis after pulmonary irradiation for metastatic Wilms’ tumor. A report from the National Wilms’ Tumor Study. Cancer.

[24] Lange J.M., Takashima J.R., Peterson S.M., Kalapurakal J.A., Green D.M., Breslow N.E. (2014). Breast cancer in female survivors of Wilms tumor: A report from the national Wilms tumor late effects study. Cancer.

[25] Wilimas J.A., Douglass E.C., Magill H.L., Fitch S., Hustu H.O. (1988). Significance of pulmonary computed tomography at diagnosis in Wilms’ tumor. J. Clin. Oncol..

[26] Meisel J.A., Guthrie K.A., Breslow N.E., Donaldson S.S., Green D.M. (1999). Significance and management of computed tomography detected pulmonary nodules: A report from the National Wilms Tumor Study Group. Int. J. Radiat. Oncol. Biol. Phys..

[27] Green D.M. (2002). Use of chest computed tomography for staging and treatment of Wilms’ tumor in children. J. Clin. Oncol..

[28] Grundy P.E., Green D.M., Dirks A.C., Berendt A.E., Breslow N.E., Anderson J.R., Dome J.S. (2012). Clinical significance of pulmonary nodules detected by CT and Not CXR in patients treated for favorable histology Wilms tumor on national Wilms tumor studies-4 and -5: A report from the Children’s Oncology Group. Pediatr. Blood Cancer.

[29] Ehrlich P.F., Hamilton T.E., Grundy P., Ritchey M., Haase G., Shamberger R.C., National Wilms’ Tumor Study Group (National Wilms’ Tumor Study 5) (2006). The value of surgery in directing therapy for patients with Wilms’ tumor with pulmonary disease. A report from the National Wilms’ Tumor Study Group (National Wilms’ Tumor Study 5). J. Pediatr. Surg..

[30] Dix D.B., Seibel N.L., Chi Y.Y., Khanna G., Gratias E., Anderson J.R., Mullen E.A., Geller J.I., Kalapurakal J.A., Paulino A.C. (2018). Treatment of Stage IV Favorable Histology Wilms Tumor with Lung Metastases: A Report from the Children’s Oncology Group AREN0533 Study. J. Clin. Oncol..

[31] Schnater J.M., Aronson D.C., Plaschkes J., Perilongo G., Brown J., Otte J.B., Brugieres L., Czauderna P., MacKinlay G., Vos A. (2002). Surgical view of the treatment of patients with hepatoblastoma: Results from the first prospective trial of the International Society of Pediatric Oncology Liver Tumor Study Group. Cancer.

[32] Meyers R.L., Katzenstein H.M., Krailo M., McGahren E.D., Malogolowkin M.H. (2007). Surgical resection of pulmonary metastatic lesions in children with hepatoblastoma. J. Pediatr. Surg..

[33] Uchiyama M., Iwafuchi M., Naito M., Yagi M., Iinuma Y., Kanada S., Tsukada K. (1999). A study of therapy for pediatric hepatoblastoma: Prevention and treatment of pulmonary metastasis. Eur. J. Pediatr. Surg..

[34] Matsunaga T., Sasaki F., Ohira M., Hashizume K., Hayashi A., Hayashi Y., Mugishima H., Ohnuma N., The Japanese Study Group for Pediatric Liver (2003). Analysis of treatment outcome for children with recurrent or metastatic hepatoblastoma. Pediatr. Surg. Int..

[35] Semeraro M., Branchereau S., Maibach R., Zsiros J., Casanova M., Brock P., Domerg C., Aronson D.C., Zimmermann A., Laithier V. (2013). Relapses in hepatoblastoma patients: Clinical characteristics and outcome—Experience of the International Childhood Liver Tumour Strategy Group (SIOPEL). Eur. J. Cancer.

[36] Shi Y., Geller J.I., Ma I.T., Chavan R.S., Masand P.M., Towbin A.J., Chintagumpala M., Nuchtern J.G., Tiao G.M., Thompson P.A. (2016). Relapsed hepatoblastoma confined to the lung is effectively treated with pulmonary metastasectomy. J. Pediatr. Surg..

[37] Temeck B.K., Wexler L.H., Steinberg S.M., McClure L.L., Horowitz M., Pass H.I. (1995). Metastasectomy for sarcomatous pediatric histologies: Results and prognostic factors. Ann. Thorac. Surg..

[38] Paulussen M., Ahrens S., Craft A.W., Dunst J., Frohlich B., Jabar S., Rube C., Winkelmann W., Wissing S., Zoubek A. (1998). Ewing’s tumors with primary lung metastases: Survival analysis of 114 (European Intergroup) Cooperative Ewing’s Sarcoma Studies patients. J. Clin. Oncol..

[39] Briccoli A., Rocca M., Ferrari S., Mercuri M., Ferrari C., Bacci G. (2004). Surgery for lung metastases in Ewing’s sarcoma of bone. Eur. J. Surg. Oncol..

[40] Letourneau P.A., Shackett B., Xiao L., Trent J., Tsao K.J., Lally K., Hayes-Jordan A. (2011). Resection of pulmonary metastases in pediatric patients with Ewing sarcoma improves survival. J. Pediatr. Surg..

[41] Raciborska A., Bilska K., Rychlowska-Pruszynska M., Duczkowski M., Duczkowska A., Drabko K., Chaber R., Sobol G., Wyrobek E., Michalak E. (2016). Management and follow-up of Ewing sarcoma patients with isolated lung metastases. J. Pediatr. Surg..

[42] Dubois S.G., London W.B., Zhang Y., Matthay K.K., Monclair T., Ambros P.F., Cohn S.L., Pearson A., Diller L. (2008). Lung metastases in neuroblastoma at initial diagnosis: A report from the International Neuroblastoma Risk Group (INRG) project. Pediatr. Blood Cancer.

[43] DuBois S.G., Kalika Y., Lukens J.N., Brodeur G.M., Seeger R.C., Atkinson J.B., Haase G.M., Black C.T., Perez C., Shimada H. (1999). Metastatic sites in stage IV and IVS neuroblastoma correlate with age, tumor biology, and survival. J. Pediatr. Hematol. Oncol..

[44] Cowie F., Corbett R., Pinkerton C.R. (1997). Lung involvement in neuroblastoma: Incidence and characteristics. Med. Pediatr. Oncol..

[45] Kammen B.F., Matthay K.K., Pacharn P., Gerbing R., Brasch R.C., Gooding C.A. (2001). Pulmonary metastases at diagnosis of neuroblastoma in pediatric patients: CT findings and prognosis. AJR Am. J. Roentgenol..

[46] Abel R.M., Brown J., Moreland B., Parikh D. (2004). Pulmonary metastasectomy for pediatric solid tumors. Pediatr. Surg. Int..

[47] Carli M., Colombatti R., Oberlin O., Bisogno G., Treuner J., Koscielniak E., Tridello G., Garaventa A., Pinkerton R., Stevens M. (2004). European intergroup studies (MMT4-89 and MMT4-91) on childhood metastatic rhabdomyosarcoma: Final results and analysis of prognostic factors. J. Clin. Oncol..

[48] Rodeberg D., Arndt C., Breneman J., Lyden E., Donaldson S., Paidas C., Andrassy R., Meyer W., Wiener E. (2005). Characteristics and outcomes of rhabdomyosarcoma patients with isolated lung metastases from IRS-IV. J. Pediatr. Surg..

[49] Weigel B.J., Lyden E., Anderson J.R., Meyer W.H., Parham D.M., Rodeberg D.A., Michalski J.M., Hawkins D.S., Arndt C.A. (2016). Intensive Multiagent Therapy, Including Dose-Compressed Cycles of Ifosfamide/Etoposide and Vincristine/Doxorubicin/Cyclophosphamide, Irinotecan, and Radiation, in Patients with High-Risk Rhabdomyosarcoma: A Report from the Children’s Oncology Group. J. Clin. Oncol..

[50] Dillon P., Maurer H., Jenkins J., Krummel T., Parham D., Webber B., Salzberg A. (1992). A prospective study of nonrhabdomyosarcoma soft tissue sarcomas in the pediatric age group. J. Pediatr. Surg..

[51] Pappo A.S., Rao B.N., Jenkins J.J., Merchant T., Poquette C.A., Cain A., Pratt C.B. (1999). Metastatic nonrhabdomyosarcomatous soft-tissue sarcomas in children and adolescents: The St. Jude Children’s Research Hospital experience. Med. Pediatr. Oncol..

[52] Kayton M.L., Meyers P., Wexler L.H., Gerald W.L., LaQuaglia M.P. (2006). Clinical presentation, treatment, and outcome of alveolar soft part sarcoma in children, adolescents, and young adults. J. Pediatr. Surg..

[53] Liu Y.P., Jin J., Wang W.H., Wang S.L., Song Y.W., Fang H., Ren H., Liu X.F., Yu Z.H., Li Y.X. (2015). A retrospective analysis of lung metastasis in 64 patients with alveolar soft part sarcoma. Clin. Transl. Oncol..

[54] Andrassy R.J., Okcu M.F., Despa S., Raney R.B. (2001). Synovial sarcoma in children: Surgical lessons from a single institution and review of the literature. J. Am. Coll. Surg..

[55] Spillane A.J., A’Hern R., Judson I.R., Fisher C., Thomas J.M. (2000). Synovial sarcoma: A clinicopathologic, staging, and prognostic assessment. J. Clin. Oncol..

[56] Stanelle E.J., Christison-Lagay E.R., Wolden S.L., Meyers P.A., La Quaglia M.P. (2013). Pulmonary metastasectomy in pediatric/adolescent patients with synovial sarcoma: An institutional review. J. Pediatr. Surg..

[57] Gulack B.C., Rialon K.L., Englum B.R., Kim J., Talbot L.J., Adibe O.O., Rice H.E., Tracy E.T. (2016). Factors associated with survival in pediatric adrenocortical carcinoma: An analysis of the National Cancer Data Base (NCDB). J. Pediatr. Surg..

[58] Schulick R.D., Brennan M.F. (1999). Long-term survival after complete resection and repeat resection in patients with adrenocortical carcinoma. Ann. Surg. Oncol..

[59] Kwauk S., Burt M. (1993). Pulmonary metastases from adrenal cortical carcinoma: Results of resection. J. Surg. Oncol..

[60] De Leon D.D., Lange B.J., Walterhouse D., Moshang T. (2002). Long-term (15 years) outcome in an infant with metastatic adrenocortical carcinoma. J. Clin. Endocrinol. Metab..

[61] Appelqvist P., Kostianinen S. (1983). Multiple thoracotomy combined with chemotherapy in metastatic adrenal cortical carcinoma: A case report and review of the literature. J. Surg. Oncol..

[62] Buddingh E.P., Anninga J.K., Versteegh M.I., Taminiau A.H., Egeler R.M., van Rijswijk C.S., Hogendoorn P.C., Lankester A.C., Gelderblom H. (2010). Prognostic factors in pulmonary metastasized high-grade osteosarcoma. Pediatr. Blood Cancer.

[63] Chou A.J., Kleinerman E.S., Krailo M.D., Chen Z., Betcher D.L., Healey J.H., Conrad E.U., Nieder M.L., Weiner M.A., Wells R.J. (2009). Addition of muramyl tripeptide to chemotherapy for patients with newly diagnosed metastatic osteosarcoma: A report from the Children’s Oncology Group. Cancer.

[64] Gelderblom H., Jinks R.C., Sydes M., Bramwell V.H., van Glabbeke M., Grimer R.J., Hogendoorn P.C., McTiernan A., Lewis I.J., Nooij M.A. (2011). Survival after recurrent osteosarcoma: Data from 3 European Osteosarcoma Intergroup (EOI) randomized controlled trials. Eur. J. Cancer.

[65] Beattie E.J., Harvey J.C., Marcove R., Martini N. (1991). Results of multiple pulmonary resections for metastatic osteogenic sarcoma after two decades. J. Surg. Oncol..

[66] Goorin A.M., Delorey M.J., Lack E.E., Gelber R.D., Price K., Cassady J.R., Levey R., Tapper D., Jaffe N., Link M. (1984). Prognostic significance of complete surgical resection of pulmonary metastases in patients with osteogenic sarcoma: Analysis of 32 patients. J. Clin. Oncol..

[67] Han M.T., Telander R.L., Pairolero P.C., Payne W.S., Gilchrist G.S., Sim F.H., Pritchard D.J. (1981). Aggressive thoracotomy for pulmonary metastatic osteogenic sarcoma in children and young adolescents. J. Pediatr. Surg..

[68] Harting M.T., Blakely M.L., Jaffe N., Cox C.S., Hayes-Jordan A., Benjamin R.S., Raymond A.K., Andrassy R.J., Lally K.P. (2006). Long-term survival after aggressive resection of pulmonary metastases among children and adolescents with osteosarcoma. J. Pediatr. Surg..

[69] Horan T.A., Santiago F.F., Araujo L.M. (2000). The benefit of pulmonary metastectomy for bone and soft tissue sarcomas. Int. Surg..

[70] Kager L., Zoubek A., Potschger U., Kastner U., Flege S., Kempf-Bielack B., Branscheid D., Kotz R., Salzer-Kuntschik M., Winkelmann W. (2003). Primary metastatic osteosarcoma: Presentation and outcome of patients treated on neoadjuvant Cooperative Osteosarcoma Study Group protocols. J. Clin. Oncol..

[71] Kempf-Bielack B., Bielack S.S., Jurgens H., Branscheid D., Berdel W.E., Exner G.U., Gobel U., Helmke K., Jundt G., Kabisch H. (2005). Osteosarcoma relapse after combined modality therapy: An analysis of unselected patients in the Cooperative Osteosarcoma Study Group (COSS). J. Clin. Oncol..

[72] Leary S.E., Wozniak A.W., Billups C.A., Wu J., McPherson V., Neel M.D., Rao B.N., Daw N.C. (2013). Survival of pediatric patients after relapsed osteosarcoma: The St. Jude Children’s Research Hospital experience. Cancer.

[73] McCarville M.B., Kaste S.C., Cain A.M., Goloubeva O., Rao B.N., Pratt C.B. (2001). Prognostic factors and imaging patterns of recurrent pulmonary nodules after thoracotomy in children with osteosarcoma. Cancer.

[74] Meyers P.A., Heller G., Healey J.H., Huvos A., Applewhite A., Sun M., LaQuaglia M. (1993). Osteogenic sarcoma with clinically detectable metastasis at initial presentation. J. Clin. Oncol..

[75] Putnam J.B., Roth J.A., Wesley M.N., Johnston M.R., Rosenberg S.A. (1983). Survival following aggressive resection of pulmonary metastases from osteogenic sarcoma: Analysis of prognostic factors. Ann. Thorac. Surg..

[76] Saeter G., Hoie J., Stenwig A.E., Johansson A.K., Hannisdal E., Solheim O.P. (1995). Systemic relapse of patients with osteogenic sarcoma. Prognostic factors for long term survival. Cancer.

[77] Slade A.D., Warneke C.L., Hughes D.P., Lally P.A., Lally K.P., Hayes-Jordan A.A., Austin M.T. (2015). Effect of concurrent metastatic disease on survival in children and adolescents undergoing lung resection for metastatic osteosarcoma. J. Pediatr. Surg..

[78] Briccoli A., Rocca M., Salone M., Guzzardella G.A., Balladelli A., Bacci G. (2010). High grade osteosarcoma of the extremities metastatic to the lung: Long-term results in 323 patients treated combining surgery and chemotherapy, 1985–2005. Surg. Oncol..

[79] Tsuchiya H., Kanazawa Y., Abdel-Wanis M.E., Asada N., Abe S., Isu K., Sugita T., Tomita K. (2002). Effect of timing of pulmonary metastases identification on prognosis of patients with osteosarcoma: The Japanese Musculoskeletal Oncology Group study. J. Clin. Oncol..

[80] Denbo J.W., Zhu L., Srivastava D., Stokes D.C., Srinivasan S., Hudson M.M., Ness K.K., Robison L.L., Neel M., Rao B. (2014). Long-term pulmonary function after metastasectomy for childhood osteosarcoma: A report from the St Jude lifetime cohort study. J. Am. Coll. Surg..

[81] Heaton T.E., Hammond W.J., Farber B.A., Pallos V., Meyers P.A., Chou A.J., Price A.P., LaQuaglia M.P. (2017). A 20-year retrospective analysis of CT-based pre-operative identification of pulmonary metastases in patients with osteosarcoma: A single-center review. J. Pediatr. Surg..

[82] Su W.T., Chewning J., Abramson S., Rosen N., Gholizadeh M., Healey J., Meyers P., La Quaglia M.P. (2004). Surgical management and outcome of osteosarcoma patients with unilateral pulmonary metastases. J. Pediatr. Surg..

[83] Karplus G., McCarville M.B., Smeltzer M.P., Spyridis G., Rao B.N., Davidoff A., Li C.S., Shochat S. (2009). Should contralateral exploratory thoracotomy be advocated for children with osteosarcoma and early unilateral pulmonary metastases?. J. Pediatr. Surg..

[84] Fernandez-Pineda I., Daw N.C., McCarville B., Emanus L.J., Rao B.N., Davidoff A.M., Shochat S.J. (2012). Patients with osteosarcoma with a single pulmonary nodule on computed tomography: A single-institution experience. J. Pediatr. Surg..

[85] Daw N.C., Chou A.J., Jaffe N., Rao B.N., Billups C.A., Rodriguez-Galindo C., Meyers P.A., Huh W.W. (2015). Recurrent osteosarcoma with a single pulmonary metastasis: A multi-institutional review. Br. J. Cancer.

